# Effect of Oral Administration of 1,3-1,6 β-Glucans in DWV Naturally Infected Newly Emerged Bees (*Apis mellifera* L.)

**DOI:** 10.3390/vetsci7020052

**Published:** 2020-04-25

**Authors:** Antonio Felicioli, Mario Forzan, Simona Sagona, Paola D’Agostino, Diego Baido, Baldassare Fronte, Maurizio Mazzei

**Affiliations:** 1 Department of Veterinary Science, University of Pisa, Viale delle Piagge 2, 56100 Pisa, Italy; antonio.felicioli@unipi.it (A.F.); mario.forzan@unipi.it (M.F.); simona.sagona@unipi.it (S.S.); p-dagostino@hotmail.it (P.D.); baldassare.fronte@unipi.it (B.F.); 2 Interdepartmental Research Center “Nutraceuticals and Food for Health”, University of Pisa, Via del Borghetto 80, 56124 Pisa, Italy; 3 Department of Pharmacy, University of Pisa, Via Bonanno 6, 56126 Pisa, Italy; 4 Independent Researcher, 37019 Peschiera del Garda (Verona), Italy; dbaido68@gmail.com

**Keywords:** β-glucans, DWV, honeybees, *Apis mellifera*, phenoloxidase

## Abstract

Honeybee pathogens have an important role in honeybee colony mortality and colony losses; most of them are widely spread and necessitate worldwide solutions to contrast honeybee’s decline. Possible accepted solutions to cope with the spread of honeybee’s pathogens are focused on the study of experimental protocols to enhance the insect’s immune defenses. Honeybee’s artificial diet capable to stimulate the immune system is a promising field of investigation as ascertained by the introduction of 1,3-1,6 β-glucans as a dietary supplement. In this work, by collecting faecal samples of honeybees exposed to different dietary conditions of 1,3-1,6 β-glucans (0.5% and 2% w/w), it has been possible to investigate the Deformed wing virus (DWV) viral load kinetic without harming the insects. Virological data obtained by a one-step TaqMan RT-PCR highlighted the ability of 1,3-1,6 β-glucans to reduce the viral load at the 24th day of rearing. The results indicated that the diet supplemented with 1,3-1,6 β-glucans was associated with a dose-dependent activation of phenoloxidase. The control group showed a higher survival rate than the experimental groups. This research confirmed 1,3-1,6 β-glucans as molecules able to modulate honeybees’ defense pathways, and this is the first report in which the kinetic of DWV infection in honeybee faeces has been monitored by a RT-qPCR.

## 1. Introduction

Deformed wing virus (DWV) is distributed worldwide and one of the most important honeybees’ pathogens, and it is responsible for the collapse of the bee colony [1,2,3]. DWV belongs to the *Picornaviridae* family within the *Iflavirus* genus [4,5]. The DWV genome consists of a positive-sense ssRNA of about 10 kb [2,5]. In overt DWV infections, honeybees show malformed or missing wings and shortened abdomens leading to premature death [2,6]. Although DWV can be mainly transmitted by the mite *Varroa destructor*, intraspecific and interspecific, vertical, and horizontal transmissions have been also demonstrated [7,8,9,10,11,12,13,14,15]. Several approaches have been proposed for controlling DWV infection [16]. A previous research indicated that β-glucans, natural molecules constituting the cell wall of many microorganisms [17,18], have antimicrobial activity in insects, by the activation of *Toll* and *Imd* signaling pathways [19]. The antiviral defense in insects is achieved mainly via RNA interference (RNAi); however, recent data suggest that the *Toll* and *Imd* pathways also contribute to defense against viral pathogens [20,21]. The response triggered by β-glucans can be remarkably different depending on the target organism and the dose administered [17,22]. Recent studies demonstrated that high concentration of 1,3 β-glucan is linked to the production of Reactive oxygen species (ROS) in carp leukocytes and the reduction of the immunostimulant effect inducing apoptosis in vitro and in vivo [23,24,25]. Caspase-3 (programmed cell death-activated endonuclease) is one of the key executioners of apoptosis [25]. Caspase-3 is a downstream effector of apoptosis that is activated by an initiator caspase [26]. Some authors observed that imidacloprid (insecticide) induced the condensation of cell nuclei and overexpression of caspase-3 in neurons, causing apoptosis in *Apis mellifera* L. [27,28]. Higes et al. [29] used the caspase-3 as indicator of apoptosis in order to investigate the effect of *Nosema* pathogenesis on honeybees [29].

Therefore, the dose of β-glucans administered is an important factor to be considered to identify the appropriate dosage for the stimulation of the immune responses. Moreover, it has been reported that the use of β-glucans as supplementary food has improved the honeybees’ immune defenses [30]. In details, western honeybees (*Apis mellifera*) fed with a diet based on different 1,3-1,6 β-glucan concentrations have been proven to induce a modification of haemocyte population and an increase of phenoloxidase (PO) activity, a key enzyme involved in pathogen encapsulation and nodule formation processes [30,31]. Phenoloxidase is expressed as prophenoloxidase (zymogen), which must be activated by serine proteinases, because some of the intermediates of the enzymatic cascade (quinones) are dangerous for the organism itself [32,33]. It has been found that in old honeybee workers the rise of PO activity compensate for the natural loss of haemocytes [34].

The PO cascade is involved in immune defense against the virus, as shown in previous studies in mosquitoes and *Pimpla turionellae* [35,36].

Furthermore, 0.5% and 2% β-glucan diets were also associated with an inhibitory effect on DWV replication [30]. 

Since information about the pharmacodynamic action of β-glucans during the experimental period were not previously considered, we planned to measure the DWV viral load from each experimental group, by collecting the honeybees’ faeces every three days for 24 days of rearing. Data on diet consumption, survival rate, phenoloxidase, and Caspase-3/CPP32 activities were also monitored, recorded, and discussed. 

## 2. Materials and Methods 

### 2.1. Rearing Conditions

A total of 390 newly emerged worker honeybees were manually collected directly from the frame in June 2017, from the experimental apiary of the Department of Veterinary Science, University of Pisa, located in San Piero a Grado (PI). 

Thirty newly emerged honeybees were collected to constitute the T0 group and stocked at −20 °C and −80 °C for enzymatic and virological assays, respectively. Additionally, 360 honeybees were sampled and randomly grouped in 12 disposable glass jars (30 individuals, each). Each jar of a volume of 750 cm^3^ (h15 cm; ∅ 8 cm) was laid on its side and equipped with a metal cap with two holes, a 1.5-cm hole for the syringe used as feeder, and another smaller hole for a 2-mL syringe used as water dispenser. There were 5 supplementary little holes done for air supply.

Honeybees were reared for 24 days in laboratory condition (at controlled temperature 28 ± 2 °C in natural dark: light cycle) and fed ad libitum with two dosages (0.5% and 2%, w/w) of 1,3-1,6 β-glucans in syrup-based diet, and with a constant supply of water. The 1,3-1,6 β-glucans (min 60%) were extracted from *Saccharomyces cerevisae* cell walls, in form of spray dried powder. The product was commercially available.

### 2.2. Feeding Conditions

A total of 12 jars with bees were prepared and submitted to three dietary conditions (4 replicates for each condition): syrup only as control diet (G_0_), syrup added with 0.5% (w/w) 1,3-1,6 β-glucans (G_0.5_), and syrup added with 2% (w/w) 1,3-1,6 β-glucans (G_2_). A commercial sugar solution containing 19% glucose, 35% fructose, 12% disaccharides, 6% trisaccharides, and 6% polysaccharides was used as control and as 1,3-1,6 β-glucan supplemented diets (Fruttosweet 45 A.D.E.A, Varese, Italy). A fresh mixture of each diet was prepared every three days to avoid any sedimentation of the β-glucans’ content. The feeder contents were checked and refilled every day.

### 2.3. Survival Rate Measurement and Food Intake Evaluation

To calculate the survival rate, dead honeybees were removed twice a day from each glass jar and counted. Dead individuals were stored at −80 °C until further analysis. Each day feeders were weighed before and after refilling to record the food consumption. Data were normalized to the number of survived honeybees present in the jar.

### 2.4. Sampling

To collect honeybees’ faeces, a piece of fan-shaped absorbent paper (0.14-mm thickness, 15.5 × 10.5 cm) was settled in each glass jar and replaced every three days. The papers collected from each replicate were transferred in disposable plastic bags and immediately processed. Faeces were harvested by making a 1-mm Ø punch on the paper. Twenty punches for each paper were pooled in a 2-mL Eppendorf and stored at –80°C, until processed for RNA extraction. 

Dead honeybees were collected every day and stored at −80 °C until RNA extraction. At day 24th, all surviving bees were sacrificed, pooled for each experimental group, and tested accordingly.

### 2.5. RNA Extraction and Viral Load Analysis

Total RNA extraction was performed on faeces, and honeybees using RNeasy mini Kit (Qiagen, Hilden, Germany). The RNA from T0 individuals, dead honeybees belonging to the control group, and from two experimental groups was extracted. Pools of three-day sampling were generated. After 24 days, all the survivor honeybees were pooled according to each experimental group and RNA extracted for viral load analysis. Briefly, punched papers with faeces or bee samples were homogenized by a stainless-bead (5 mm Ø) in a Tissue Lyser II (Qiagen, Hilden, Germany). Total RNA was eluted in 30 μL RNase-free water and quantified by a Qubit RNA HS kit by the Qubit fluorometer (Thermo fisher scientific, Waltham, MA, USA). Five microliters of each extracted RNA were then used as template to determine the viral load by one-step TaqMan RT-PCR assay [9]. The results were normalized for the number of bees in the cage expressed as viral copy number per microgram of input RNA for bee sample.

### 2.6. Phenoloxidase Activity 

Six honeybees from each replicate (24 honeybees per control and experimental groups) were sampled at 0 and at 24th day of rearing to measure the PO activity. Each bee head was weighed and soaked in 200 µL of 50 mM phosphate buffer pH 7.2 with 1% Triton X-100, left at −20°C for 20 min and homogenized by a Teflon pestle. Centrifugation at 4000 rpm, 4 °C, for 15 min was performed and the supernatant collected. Protein concentration was measured by Qubit Protein Assay Kit by the Qubit fluorometer (Thermo Fisher Scientific, Waltham, MA, USA). A PO assay was performed by a modified Alaux protocol [30,37]. Absorbance data were obtained at λ = 490 nm for 10 min. PO values were expressed as UE/mg of tissue.

### 2.7. Caspase-3 Activity Assay

Caspase-3 activity was measured by the Caspase-3/CPP32 colourimetric Assay Kit (BioVision, Mountain View, CA, USA). The assay is based on the spectrophotometric detection of the chromophore p-nitroaniline (pNA) after cleavage from the labelled N-Acetyl-Asp-Glu-Val-Asp p-nitroanilide substrate (DEVD-pNA). Two pools of three abdomens for each treatment (G_0_, G_0.5_, and G_2_) were tested. Briefly, the abdomens were mixed with 300 μL of PBS buffer pH 7.4, homogenized by a Teflon pestle and, after a macro filtration, centrifuged at 14000 rpm for 4 min. Pellets were lysed in 100 μL of lysis buffer and incubated on ice for 10 min. After centrifugation at 13000 rpm for 1 min, supernatants (extracts) were collected, and the protein content quantified by Qubit fluorometer (Thermo Fisher Scientific, Waltham, MA, USA). The extracts were diluted in lysis buffer in order to obtain the concentration of 1 μg/μL of total proteins. Fifty μL of a 2X reaction buffer (containing 10 mM DTT) and 5 µL of the 4 mM DEVD-pNA substrate were added to each extract (50 µL) and incubate at 37 °C for 1 h. Reactions were measured at 405 nm in a microtiter plate reader. 

### 2.8. Statistical Analysis 

Statistical analysis was performed using the JMP© software (SAS Institute, Cary, NC, USA, 2008). The estimated survival rate was analyzed by Log–rank (Mantel–Cox) test. When factors significantly differed from homogeneous distribution, paired tests were performed. Food intake, viral load, Caspase-3 assay, and PO analysis were performed by the following criteria: data residues obtained by preliminary ANOVA for more factors were tested for normal distribution by Shapiro–Wilk test. When their distribution differed significantly from the normal distribution, these data were analyzed by nonparametric Kruskal–Wallis test, while when values were in accord with a normal distribution, the data were analyzed by Student *t*-test. 

## 3. Results

### 3.1. Survival Rate

**Figure 1:**
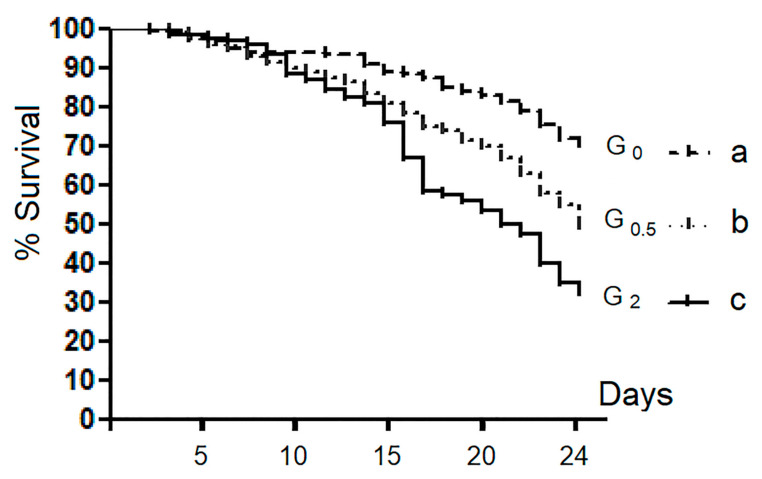
Survival curves: comparison among groups. Each curve indicates the sum of four replicates for the group. In G_0_, G_0.5_, and G_2_, are grouped bees fed with 0%, 0.5%, and 2% of 1,3-1,6 β-glucans (w/w) in syrup, respectively. Different letters show statistically significant differences (*p* < 0.01).

Survival rate analysis showed statistical differences among all experimental groups (*p* < 0.001) (Figure 1). 

At the 24th day of rearing, the three experimental groups scored survival values significantly different (*p* < 0.01), with the G_2_ (32.5%) and G_0_ (70.3%) groups showing the lowest and the highest survival values, respectively. 

### 3.2. Food Intake

The mean daily syrup consumption for the control group and the two experimental groups measured during the whole experimental period resulted in G_0_ 19.9 ± 0.7 mg, G_0.5_ 19.7 ± 0.8 mg, and G_2_ 20.9 ± 0.8 mg. No statistical differences were observed among groups. 

### 3.3. Viral Loads

#### 3.3.1. Viral Load of the Faecal Samples

**Figure 2:**
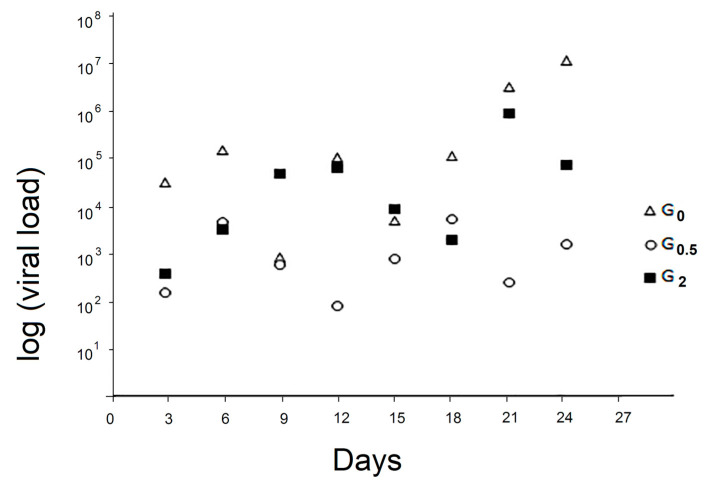
Viral load in the faecal samples. In G_0_, G_0.5_, and G_2_, are grouped bees fed with 0%, 0.5%, and 2% of 1,3-1,6 β-glucans (w/w) in syrup, respectively. Values were expressed as exponential function.

The kinetics of viral load of the faecal samples of the control group and the two experimental groups are reported in Figure 2. 

No significant differences were identified between the two experimental groups and the control one; only at day 24, G_0_ resulted as significantly higher than G_0.5_ (*p* < 0.05). 

#### 3.3.2. Viral Load of Whole T0 Honeybee Samples

The mean viral load of T0 samples was 2.28 × 10^2^ (SD 0.72 × 10^2^).

#### 3.3.3. Viral Load of the Whole Dead Honeybee Samples

No statistical differences were observed for the viral loads of dead honeybees collected during the experiment. The median for G_0_, G_0.5_, and G_2_ viral load values were 1.05 × 10^5^, 1.09 × 10^4^, and 8.28 × 10^3^, respectively. 

#### 3.3.4. Viral Load of the Whole Survived Bee Samples

**Figure 3:**
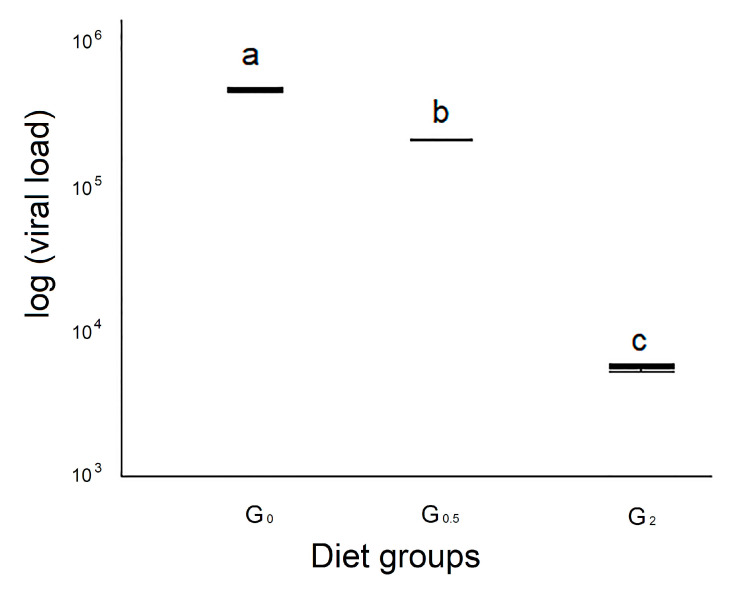
Viral load in the whole bee samples. In G_0_, G_0.5_, and G_2_, are grouped bees fed with 0%, 0.5%, and 2% of 1,3-1,6 β-glucans (w/w) in syrup, respectively. Values were expressed as exponential function. Different letters show statistically significant differences (*p* < 0.05).

The viral load of the survived honeybees analyzed at the 25th day belonging to group G_0_ showed the highest median viral load (4.90 × 10^5^/copies DNA microgram), an intermediate value (2.22 × 10^5^/copies DNA microgram) for group G_0.5_, and the lowest value was observed for group G_2_ (5.86 × 10^3^), statistically different from the control group and the other experimental one (*p* < 0.01) (Figure 3). 

### 3.4. Phenoloxidase Activity

**Figure 4:**
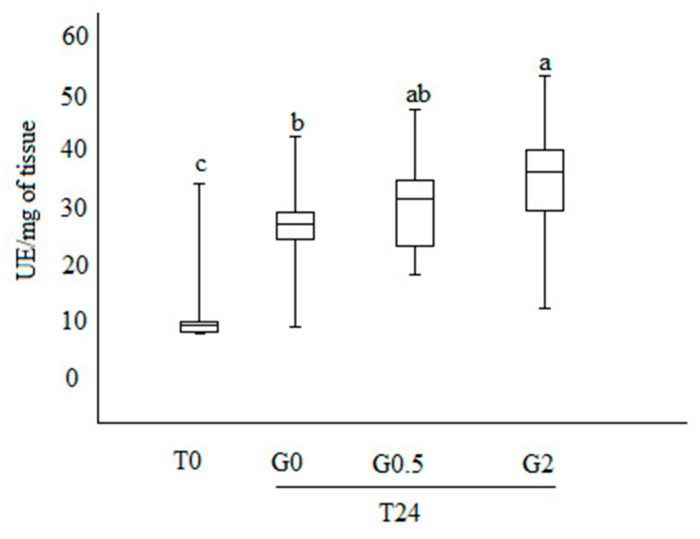
Boxplot representing phenoloxidase activity (expressed in UE/mg of tissue). T0, bees stored at the time of the sample collection; T24, bees after 24 days of rearing; G_0_, G_0.5_, and G_2_, bees fed with 0%, 0.5%, and 2% of 1,3-1,6 β-glucans (w/w) in syrup, respectively. Different letters show statistically significant differences (*p* < 0.01).

The PO activity observed at T0 was lower than that observed after 24 days of trial, independently from the diet administered (*p* < 0.01) (Figure 4).

On day 24th, group G_2_ showed the highest PO activity (34.6 ± 1.8 UE/mg of tissue) and G_0_ the lowest (27.2 ± 1.3 UE/mg of tissue) (*p* < 0.01). G_0.5_ showed an intermediate PO value (30.3 ± 1.6 UE/mg of tissue), not significantly different from both the other analyzed groups (*p* > 0.05). 

### 3.5. Caspase-3 Activity Assay

The Caspase-3 analysis showed no significant differences among treatments (*p* > 0.05). In particular, the mean of the absorbance values at 405 nm were 0.230 ± 0.036, 0.270 ± 0.023, and 0.168 ± 0.073, for G_0_, G_0.5_, and G_2_, respectively. 

## 4. Discussion

The administration of 1,3-1,6 β-glucans to DWV infected honeybees reduces DWV viral load if compared with the control group. This result is linked to immune parameters’ modulation as phenoloxidase activity and haemocyte count, as previously reported [30]. In the present study, to investigate the effects of a continuous oral administration of 1,3-1,6 β-glucans, honeybees reared in experimental condition were fed with diets supplemented with different concentrations of 1,3-1,6 β-glucans during a period of 24 days. The newly emerged honeybees resulted infected, as indicated by a low viral load recorded for T0 samples. This evidence allowed us to perform the experiment without inducing the infection experimentally. Moreover, the method of rearing using glass jars allowed us to conduct the study using sterile material due the possibility of autoclaving all the parts. Furthermore, this system permits to observe the honeybees’ vitality, easily replace the paper, and collect the dead honeybees. The fan-paper shape was useful to obtain a wider surface to collect feaces.

The survival rate of honeybees belonging to the control group (G_0_) showed values comparable with those indicated by previous studies performed in honeybees reared in similar conditions [38,39]. The results obtained in this investigation indicate that the presence of 1,3-1,6 β-glucans in the syrup diet had a negative dose-dependent effect on the survival rate. The diet containing 2% and 0.5% β-glucans supplement caused a lower survival rate in comparison with the control diet. This result suggests a possible dose-dependent toxic effect due to the β-glucan administration that could induce a prolonged stimulation of the immune defense system, resulting in a negative effect of metabolic intermediate accumulation, as previously reported in invertebrates and in insects by Söderhäll and Cerenius [40] and González-Santoyo and Córdoba-Aguilar [31], respectively. 

No statistical differences were observed on the food intake among control bees and the two experimental groups, indicating that 1,3-1,6 β-glucans do not determine lower or higher attractiveness than only syrup diet. Caspase-3 activity was not different among groups. Since caspase is an indicator of apoptosis [25], this result indicated that β-glucans administered as supplementary diet to honeybees do not induce apoptosis. Previous studies on apoptosis were performed on fish using 1,3 β-glucans instead of 1,3-1,6 β-glucans; those elements could be the reason for the differences on apoptosis obtained in honeybees [23,24].

A significant difference in viral loads in faecal samples was detected only at the 24th day, only between G_0_ and G_0.5_. To the best of our knowledge, since faecal excretion of the virus from infected honeybees has been previously reported [11], this is the first report in which the kinetic of DWV infection in honeybees’ faeces has been monitored by a RT-qPCR. Although the lack of statistically significant differences between DWV levels in faeces produced by bees with different levels of DWV was observed, the detection of DWV in faecal honeybee samples is potentially useful as a way of non-invasive DWV level monitoring. Further investigations are desirable to improve this method.

No statistical differences were observed for the viral loads of dead honeybees collected during the course of the experiment. Noteworthy, the viral load of survived honeybees belonging to G_0.5_ and G_2_ was lower than in the control group. This result strongly suggests a relationship between 1,3-1,6 β-glucans and DWV. The highest values of PO at day 24 scored by honeybees fed with 2% β-glucans could be due to the well-known ability of 1,3-1,6 β-glucans to activate the proteases involved in activating phenoloxidase, as reported by Vetvicka and Sima [41] in invertebrates, which, in turn, is involved in melanisation of pathogens. The main role of PO in melanogenesis is to convert phenols to quinones, which subsequently polymerize to form melanin, resulting in deposition around the damaged tissue or foreign objects. The melanin capsule prevents the growth and reproduction of the pathogen and eventually leads to its death, mainly by starvation.

When we compared viral load values with PO activity on the surviving bees, the G_0_ group scored the highest viral load and the lowest PO activity, while G_2_ showed the lowest viral load and the highest PO activity. G_0.5_ showed intermediate values for both parameters. Such results could be interpreted as a 1,3-1,6 β-glucan dose-dependent activation of PO that results in a viral replication restraint. 

In order to explain the relationship between survival rate and viral loads, we can take in consideration three hypotheses: (i) Surviving honeybees fed with 0.5%–2% 1,3-1,6 β-glucans could be a selected population resistant to the treatment, (ii) surviving honeybees are individuals with a low rate of virus replication due to 0.5%–2% 1,3-1,6 β-glucans feeding, and (iii) the combination of both of the above factors. 

If the first hypothesis is considered, we would expect a lower viral load in the dead honeybees fed with 1,3-1,6 β-glucans compared with the control honeybees, due to an additional negative effect of the diet on viral pathogenesis. Since the viral load in dead honeybees among groups did not differ, this result is in accordance with the second hypothesis. Considering the survival rate alone, 1,3-1,6 β-glucans decreased the honeybees’ survival, but independently from the individual viral load. This suggests that there was not an effect on the selection of surviving honeybees, but there was an effect of 1,3-1,6 β-glucans on the viral replication. 

In conclusion, the role of β-glucans as immune-modulator molecules has been reported for several species such as poultry, swine, and fish [42,43,44]. This research indicates that 1,3-1,6 β-glucans administered as supplementary food have a role on modulating the honeybee’s defense pathways. To better characterize and define the 1,3-1,6 β-glucans’ effects on honeybees, additional studies are necessary, in order to define their posology and method of administration. 

Since the use of 1,3-1,6 β-glucans results in an effective tool to modulate the immune system against viral pathogens, the same dietary approach could be applied to increase the natural immune defenses against other diffuse honeybee pathogens, such as bacteria, and gut microorganisms, such as *Crithidia mellificae* or *Nosema* spp.
